# Early warning of citric acid overdose and timely adjustment of regional citrate anticoagulation based on machine learning methods

**DOI:** 10.1186/s12911-021-01489-8

**Published:** 2021-07-30

**Authors:** Huan Chen, Yingying Ma, Na Hong, Hao Wang, Longxiang Su, Chun Liu, Jie He, Huizhen Jiang, Yun Long, Weiguo Zhu

**Affiliations:** 1 Department of Critical Care Medicine, State Key Laboratory of Complex Severe and Rare Diseases, Peking Union Medical College Hospital, Peking Union Medical College, Chinese Academy of Medical Sciences, Beijing, 100730 China; 2 Digital Health China Technologies Co., Ltd., Beijing, 100080 China; 3 Department of Primary Care and Family Medicine, State Key Laboratory of Complex Severe and Rare Diseases, Peking Union Medical College Hospital, Peking Union Medical College, Chinese Academy of Medical Sciences, Beijing, 100730 China; 4 Department of General Internal Medicine, Department of Information Center, State Key Laboratory of Complex Severe and Rare Diseases, Peking Union Medical College Hospital, Peking Union Medical College, Chinese Academy of Medical Sciences, Beijing, 100730 China

**Keywords:** Anticoagulants, Continuous renal replacement therapy, Machine learning, Intensive care units

## Abstract

**Background:**

Regional citrate anticoagulation (RCA) is an important local anticoagulation method during bedside continuous renal replacement therapy. To improve patient safety and achieve computer assisted dose monitoring and control, we took intensive care units patients into cohort and aiming at developing a data-driven machine learning model to give early warning of citric acid overdose and provide adjustment suggestions on citrate pumping rate and 10% calcium gluconate input rate for RCA treatment.

**Methods:**

Patient age, gender, pumped citric acid dose value, 5% NaHCO_3_ solvent, replacement fluid solvent, body temperature value, and replacement fluid PH value as clinical features, models attempted to classify patients who received regional citrate anticoagulation into correct outcome category. Four models, Adaboost, XGBoost, support vector machine (SVM) and shallow neural network, were compared on the performance of predicting outcomes. Prediction results were evaluated using accuracy, precision, recall and F1-score.

**Results:**

For classifying patients at the early stages of citric acid treatment, the accuracy of neutral networks model is higher than Adaboost, XGBoost and SVM, the F1-score of shallow neutral networks (90.77%) is overall outperformed than other models (88.40%, 82.17% and 88.96% for Adaboost, XGBoost and SVM). Extended experiment and validation were further conducted using the MIMIC-III database, the F1-scores for shallow neutral networks, Adaboost, XGBoost and SVM are 80.00%, 80.46%, 80.37% and 78.90%, the AUCs are 0.8638, 0.8086, 0.8466 and 0.7919 respectively.

**Conclusion:**

The results of this study demonstrated the feasibility and performance of machine learning methods for monitoring and adjusting local regional citrate anticoagulation, and further provide decision-making recommendations to clinicians point-of-care.

**Supplementary Information:**

The online version contains supplementary material available at 10.1186/s12911-021-01489-8.

## Background

With the increasingly widespread use of continuous renal replacement therapy (CRRT) in critically ill patients, choosing a safe, effective, sustained, and stable anticoagulation approach is very important for intensive care units (ICU) practitioners. Multiple organ failure, surgical trauma, active bleeding, disseminated intravascular coagulation, etc. are common in critically ill patients. Heparin has been widely used for anticoagulation treatments owing to its good anticoagulant effect and low price [1]; however, improper heparin application increases the risk of bleeding, leading to heparin-related thrombocytopenia, bleeding, or other adverse outcomes. Moreover, this is likely to delay the detection of the anticoagulation level, reduce filter life and increase cost [2]. Local regional citrate anticoagulation provides good cardiopulmonary bypass anticoagulation, fewer bleeding complications, and improved filtration membrane biocompatibility [3, 4], has gradually replaced heparin as the first-line local anticoagulation strategy in CRRT process in guidelines such as the Kidney Disease Improving Global Outcomes (KDIGO) Clinical Practice Guidelines [5–7].

Local citrate/calcium antagonism is an important local anticoagulation method during bedside renal replacement therapy. The method involves using citrate to chelate ionized calcium in the blood in vitro to achieve anticoagulation, and passing it through the vein pathway pumps. The body self-metabolizes the citrate-calcium to supplement the lost calcium ions. During treatment, it is necessary to repeatedly monitor the ionized calcium before and after the filter [i.e., the ionized calcium level after regional citrate anticoagulation (RCA)] to detect effective hypocalcemia, as well as monitor the total blood calcium (i.e., the total calcium level after supplementation in the body), for timely adjustment of the citrate level to ensure safety [8–10].

Local regional citrate anticoagulation has been proven to have an effective in vitro anticoagulation effect and can be used to avoid prolonged heparin exposure and heparin accumulation during CRRT involving heparin anticoagulation [4–8]. The advantages of local regional citrate anticoagulation include reduction in dialysis-related bleeding complications, improvement in the permeability of the dialyzer, improvement in the biocompatibility of the CRRT, and prolongation of the service life of the filter. In addition, citrate anticoagulation abolishes degranulation of polymorphonuclear cells and platelets and reduces oxidative stress during hemodialysis [11–14]. Therefore, it has been used in the treatment of critically ill patients with acute kidney injuries. However, the lack of dose monitoring in RCA treatment can still cause serious complications during RCA, such as metabolic alkalosis and acidosis, hypernatremia, hypocalcemia, or hypercalcemia, which are caused by citrate accumulation [15, 16]. Insufficient anticoagulation will cause frequent coagulation, and thrombosis will decrease the functionality of the filter, leading to a serious shortening of the effective treatment time of hemofiltration. This not only increases the cost of treatment but also causes the loss of additional blood components. Excessive anticoagulation can lead to citric acid overdose and can endanger the life of the patient.

Furthermore, citrate mainly binds to calcium and to a lesser extent with other cations (such as magnesium). A citric acid overdose mainly occurs when the filtration increases the loss of the calcium combined with the citric acid. The implementation of an effective and safe local regional citrate anticoagulation regimen depends largely on rapid regulation of blood calcium levels in the body, as hypocalcemia directly threatens the life of the patient and hypercalcemia can cause anticoagulation failure. Controlling the citric acid intake pump is very important, and there is an urgent need for standardized clinical implementation in regional citrate anticoagulation. Therefore, to avoid the occurrence of adverse reactions, it is necessary to monitor the concentration of ionized calcium in the body, and timely adjust the calcium supplementation rate [17]. In recent years, with the rapid development of machine learning technologies, data-driven methods has been introduced in this fields to provide algorithm generated outcome predictions and dosing related clinical decision supports for clinicians [18, 19]. These previous studies demonstrated that by closely monitoring the bedside patient data, it is possible to predict treatment outcomes, and to provide computer generated recommendations.

The purpose of this study was to predict the ranges of post-filter ionized calcium levels which reflect the treatment outcomes of local regional citrate anticoagulation based on machine learning methods. By further closely monitoring the ratio of total calcium measured in vitro and free calcium measured in blood gas, this study is to early identify whether the patient is experiencing citric acid overdosing, and to provide timely adjustment suggestions on citrate pumping rate and 10% calcium gluconate input rate to clinicians point-of-care.

## Methods

### Study design overview

**Fig. 1:**
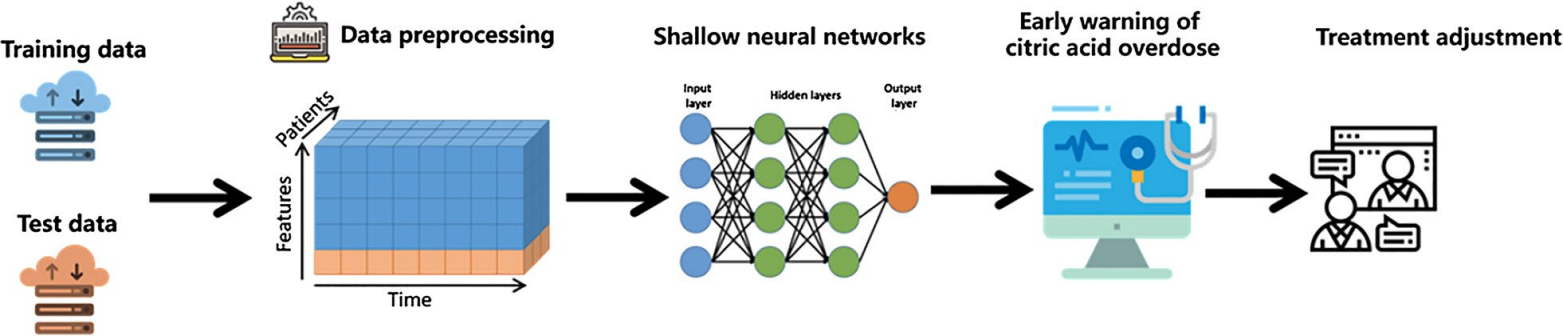
The machine learning based early warning and adjustment for local regional citrate anticoagulation therapy

The overall design of this study was shown as Fig. 1. A machine learning based early warning and adjustment method was developed and evaluated for local regional citrate anticoagulation therapy. Early warning of citric acid overdosing was achieved through predicting the ranges of post-filter ionized calcium levels which reflect the treatment outcomes by using machine learning models, such as shallow neural networks. Accordingly, timely adjustment suggestions were provided to clinicians point-of-care.

#### Outcome

The primary outcome was post-filter ionized calcium level. As the post-filter ionized calcium in the blood gas will change after 4 h when pumping citric acid for patients, the post-filter ionized calcium concentration 4 h after the citric acid pumping time were selected and labeled as the classification targets for this treatment outcome prediction task. Based on current practical implementation guidelines for citric acid anticoagulant renal replacement therapy, the post-filter ionized calcium values from common blood gas analyzers were divided into four ranges: < 0.25 mmol/L, 0.25–0.35 mmol/L, 0.35–0.5 mmol/L, and > 0.5 mmol/L.

#### Study population

Our study used the ICU patient database of Peking Union Medical College Hospital (PUMCH) to identify patients with citric acid overdose. The PUMCH ICU database comprises the complete clinical data of patients admitted to the PUMCH ICU with a retrospective cohort of totally 20,778 ICU patients during 2013–2019. For model development, testing and validation, we divided above PUMCH ICU patient data into two datasets. The dataset 1 with patient data from 2013 to 2018 used for model development and test, and to validate the predicative ability of established models using an external and recent cohort, the dataset 2 with patient data of 2019 was extracted for validation.

**Fig. 2:**
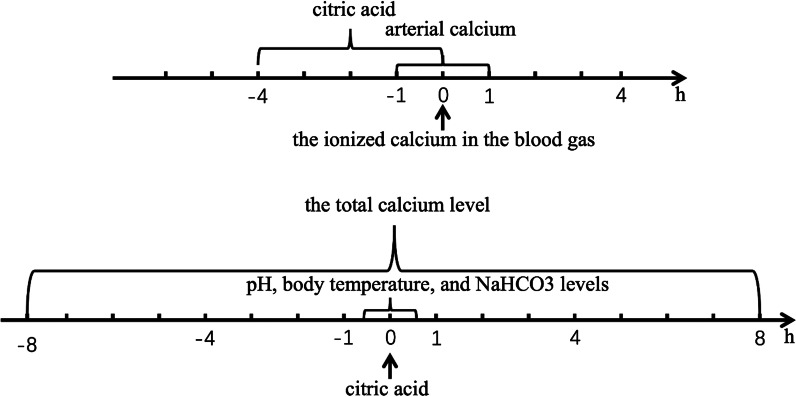
Patient data selection event timeline

We enrolled patient data from PUMCH ICU database in accordance with the following rules. All the adult patients that above 18 years old and received regional citrate anticoagulation therapy during their ICU stay were included. Based on the measurement time of the ionized calcium in the blood gas, the blood gas arterial calcium within 1 h and citric acid before 4 h were then extracted. We further obtained the measurement values of pH, body temperature, and NaHCO_3_ levels within 0.5 h before and after of citric acid pumping time, as well as the total calcium level within 8 h before and after the measurement time of ionized calcium in the blood gas, as shown in Fig. 2. As one patient has more than one observed record during their citrate treatment, we then randomly selected one record from each patient that with different values of post-filter ionized calcium. In total, 1503 records from 312 patients were included in dataset 1 and 431 records from 81 patients were included in dataset 2.

#### Feature selection

Regional citrate anticoagulation can be applied in various CRRT modes, including continuous venous–venous hemofiltration, continuous venous–venous hemodialysis, and continuous venous–venous hemodialysis. Moreover, anti-coagulation regimens are complex and diverse, and the regimens at different institutions are somewhat different, based on their own conditions. At present, there are two commonly used methods. One is the pre-filled method, which uses citric acid as a part of a replacement fluid, to simultaneously provide anticoagulation and bases, and to provide replenishment before the input of replacement fluid. This method is simple, but the changes in blood flow and replacement fluid will affect the anticoagulant effect. The second method is peripheral pumping, which is relatively complicated. The peripheral pumping method ensures the full anticoagulation of the entire dialysis line, which is convenient for adjusting and monitoring the dosage of citrate, but it is more prone to causing metabolic complications. Therefore, the replacement fluid-related factors and esterification fluid-related factors were selected as the input features. In addition, as selection of the regional citrate anticoagulation regimen also relies on the concentration of citrate, the factors that affect the post-filer ionized calcium concentration were further included as input features. According to the above related factors analysis, patient age, gender, pumped citric acid dose value, 5% NaHCO_3_ solvent, replacement fluid solvent, body temperature value, and replacement fluid PH value were selected as the input features.

#### Data preprocessing

The PUMCH ICU database was well-curated with very few (less than 3%) missing data of our analysis datasets. For the occasional missing values of patient temperature and replacement fluid, a mean substitution method was used for imputation of the missing data. After the missing data were filled in, the mean value of the variable remained unchanged. The data were normalized using min–max normalization method as follows:

$${X}^{*}=({X}_{i}-{X}_{min})/({X}_{max}-{X}_{min})$$

where ${X}_{i}$ is the original value of feature i and ${X}_{min}$ and ${\mathrm{X}}_{max}$ represent the statistical minimum value and maximum value of the Feature i, respectively [20].

### Predictive modeling for citric acid overdose

#### Machine learning models

The pumping speed of the citric acid affects the concentration of the post-filter ionized calcium in the blood during citric acid pumping. We performed four binary classification tasks for each category to predict the concentrations of post-filter ionized calcium in the blood at the early stages of treatment [21]. Four machine learning models, i.e., the Adaboost, XGBoost, support vector machine (SVM), and shallow neural network, were selected for predicting these four binary classification tasks. Both AdaBoost and XGBoost are based on using a boost as a learning method. This method selects known features to improve the predictive ability of the model, thereby reducing its dimensionality. The feature of a sample is the output of the weak classifier applied to each sample. AdaBoost trains different weak classifiers by changing the weights of samples and combines the weak classifiers into a weighted sum representing the final output of the enhanced classifier [22] and XGBoost is a scalable tree boosting system that based on gradient enhancement [23]. The algorithm learns a set of enhancement trees and makes a careful trade-off between classification errors and model complexity. XGBoost recently dominated a competition regarding applied machine learning and Kaggle-structured or tabular data. The SVM is based on maximizing the boundary between the two types of data, i.e., maximizing the minimum distance to the nearest sample separating the hyperplane. The Gaussian kernel ensures that the classification is non-linear. The neural network constructs multiple layers of neurons. Each neuron receives a large number of input variables and passes the results to the next layer [24]. A shallow neural network can learn complex functions related to input and output variables, and can handle variables, complex functions, and complex relationships. The shallow neural network is built using TensorFlow 1.13.1. The key parameters of the four models were listed in Additional file 1: Appendix I.

We used the GridSearch function in the scikit-learn package to search for the best parameters of the SVM, AdaBoost, and XGBoost algorithms, and then used the best parameters to train the model. The shallow neural network model contains two hidden layers, each containing 36/24 neurons, and we used a modified linear function as the activation function. The output layer is category number 2, and the loss function is the cross-entropy loss function.

Among the results was the one-hot label of the sample, i.e., the probability of the output calcium type of the sample after passing through the network. A batch gradient descent algorithm was adopted in the training process, and the batch size was 500. The network training used dropout to randomly discard neurons, thereby enhancing the generalization ability of the model. The dropout drop rate is 0.75; an early stop setting was used, and a regular term is added to the loss function to prevent overfitting. The initial learning rate is 0.0015, and a total of 3000 rounds were trained.

To train and test their prediction performances, a fivefold cross-validation method was used for Adaboost, XGBoost, SVM and shallow neural network, respectively. The dataset 1 was randomly divided into 5 equal parts, one of which was taken as testing set each time; the remaining four were used as the training set. The training was performed five times for each model, and the comprehensive performance of each model was evaluated.

#### Model performance evaluation and validation

To evaluate the model performance in the classification of post-filter ionized calcium, we used the precision, recall, F1-score, and accuracy as our evaluation indicators. Precision denotes the proportion of false positives. Recall measures false negatives against true positives. The F1-score is the harmonic average of the precision and recall. Accuracy is the proportion of correct predictions over the output results. For all samples of each label, the micro-averaged precision, recall, and F1 score are all equal to the accuracy; therefore, we measure the classification performance of these models by comparing the average accuracy of the models on a macro level. Furthermore, we used the LASSO to study the features. LASSO is a shrinkage estimation method. By regressing and penalizing all variables, the coefficients of relatively unimportant variables become 0 and they are excluded from modeling, and then the independent variables that have a greater impact on the dependent variable are selected and calculated. To validate the model generalizability, we chose the model with the best comprehensive performance as the implementation model for external validation using dataset 2. To further evaluate the capability of machine learning models for citric acid overdose predictive modeling problem, we conducted an extended experiments and evaluation using The Multiparameter Intelligent Monitoring in Intensive Care (MIMIC) III database. The modeling implementations and results are listed in Additional file 1: Appendix II.

#### Determination method of citric acid overdose

A citric acid overdose can occur in cases of liver dysfunction and citric acid infusion, after prolonged renal replacement therapy exceeds the human metabolic capacity. A citric acid overdose can be detected by closely monitoring the total calcium level and post-filter ionized calcium level. The measurement time for the total calcium in the renal total test is every 2 h in the first 8 h after hemofiltration, and every 4 h in the next 16 h. If the patient is stable, monitoring is conducted once every 6–8 h. When the ratio of the total calcium/ionized calcium increases, a value greater than 2.5 indicates an overdose.

### Early warning and adjustment by close monitoring of patient data

We further assessed the citric acid overdose incidence proportion among different post-filter ionized calcium levels groups using the PUMCH ICU dataset. The assessment results may provide early warning and guide clinicians in implementing effective monitoring for different patient groups.

To achieve the desired level of citric acid anticoagulation by timely controlling and adjusting the citrate pumping speed and calcium supplementation rate at the point of care, two adjustment suggestions for regional citrate anticoagulation therapy are provided. These suggestions are based on the abovementioned prediction results obtained with the close monitoring of patient data, and they comprise: (1) adjustment suggestions regarding the citrate pumping rate to maintain an appropriate post-filter ionized calcium level; (2) adjustment suggestions regarding the pumping of 10% calcium gluconate if the patient has citric acid poisoning.

## Results

### Summary statistics

Based on classification, the collected PUMCH ICU patient data were divided into four categories. Summary statistics of selected features from PUMCH ICU dataset are listed in Table 1.

**Table 1:** Summary Statistics of PUMCH ICU dataset

Features	Outcomes (post-filter ionized calcium levels)			
	< 0.25 mmol/L	0.25–0.35 mmol/L	0.35–0.5 mmol/L	> 0.5 mmol/L
*Dataset 1: PUMCH (n = 1503)*	84	379	802	238
Age	62.98 (15.06)	59.65 (15.14)	56.01 (15.89)	56.86 (15.56)
Temperature	36.64 (0.62)	36.58 (0.62)	36.61 (0.67)	36.58 (0.70)
Replacement fluid pH value	7.21 (0.17)	7.25 (0.12)	7.27 (0.09)	7.29 (0.09)
Value of citrate	207.94 (82.20)	201.86 (34.90)	201.64 (52.70)	169.49 (60.35)
5% NaHCO_3_	75.41 (31.47)	75.50 (32.38)	73.95 (34.07)	83.43 (38.06)
Replacement fluid	1880.82 (1234.24)	1879.92 (929.15)	1820.23 (740.11)	1685.53 (509.19)
Gender, n (%)				
Male	67.86 (%)	63.06 (%)	65.84 (%)	65.13 (%)
Female	32.14 (%)	36.94 (%)	34.16 (%)	34.87 (%)
*Dataset 2: PUMCH (n = 431)*	20	100	219	92
Age	58.50 (15.83)	60.67 (14.68)	59.39 (15.14)	63.00 (12.56)
Temperature	36.43 (0.65)	36.41 (0.56)	36.40 (0.66)	36.41 (0.63)
Replacement fluid PH value	7.36 (0.07)	7.36 (0.07)	7.36 (0.06)	7.35 (0.07)
Value of citrate	196.48 (77.69)	185.27 (84.73)	172.48 (60.07)	132.83 (42.09)
5% NaHCO_3_	41.79 (31.09)	48.05 (34.92)	53.57 (36.02)	66.25 (43.49)
Replacement fluid	1770.55 (645.64)	1850.79 (974.67)	1698.43 (543.06)	1649.49 (820.87)
Gender, n (%)				
Male	65.00 (%)	73.00 (%)	64.38 (%)	40.22 (%)
Female	35.00 (%)	27.00 (%)	35.62 (%)	59.78 (%)

### Model prediction results

We dichotomy each label and select all samples in each category, where the number of samples corresponding to label 0, 1, 2 and 3 are respectively: 84,379,802 and 238. For dataset 1, we have a total of 1503 positive samples. The model performance results are shown in Table 2.

**Table 2:** Model performances for predicting post-filter ionized calcium levels

Labels	Models	Precision (%)	Recall (%)	F1-score (%)	Accuracy (%)
“0”: < 0.25 mmol/L	AdaBoost	70.43	69.61	69.99	77.94
	XGBoost	83.73	79.41	81.20	86.76
	SVM	**91.13**	67.65	71.22	83.82
	Shallow neural network	90.76	**90.77**	**90.77**	**90.76**
“1”: 0.25–0.35 mmol/L	AdaBoost	67.39	59.21	60.04	76.32
	XGBoost	82.65	78.54	86.41	85.17
	SVM	**92.93**	77.41	79.72	86.25
	Shallow neural network	88.45	**88.4**	**88.40**	**88.45**
“2”: 0.35–0.5 mmol/L	AdaBoost	70.22	59.11	59.85	77.17
	XGBoost	**83.77**	**80.92**	**82.17**	83.77
	SVM	81.07	80.89	81.87	81.89
	Shallow neural network	83.74	**80.92**	**82.17**	**88.77**
“3”: > 0.5 mmol/L	AdaBoost	73.15	68.40	70.03	79.69
	XGBoost	83.91	81.94	82.85	87.50
	SVM	**91.38**	68.75	72.56	84.38
	Shallow neural network	88.98	**88.96**	**88.96**	**88.96**

The bold means the best performed model for each evaluation indicator

The F1 score represents a comprehensive evaluation of the model performance. As listed in Table 2, extreme gradient boosting achieved the second best F1 score (81.20%, 86.41%, 82.17%, and 82.85% for labels 0, 1, 2, and 3 of the PUMCH ICU database, respectively), second only to that of shallow neural network (90.77%, 88.40%, 82.17%, and 88.96% for labels 0, 1, 2, and 3 of the PUMCH ICU database, respectively). The SVM model also performed very well for all three data sets (71.22%, 79.72%, 81.87%, and 72.56% for labels 0, 1, 2, and 3 of the PUMCH ICU database, respectively). The adaptive boosting model performed slightly worse (69.99%, 60.04%, 59.85%, and 70.03% for labels 0, 1, 2, and 3 of the PUMCH ICU database, respectively) than the above three models. Therefore, we recommend shallow neural network as the practical classifier model for predicting post-filter ionized calcium levels using close monitoring of patient data during regional citrate anticoagulation therapy.

**Fig. 3:**
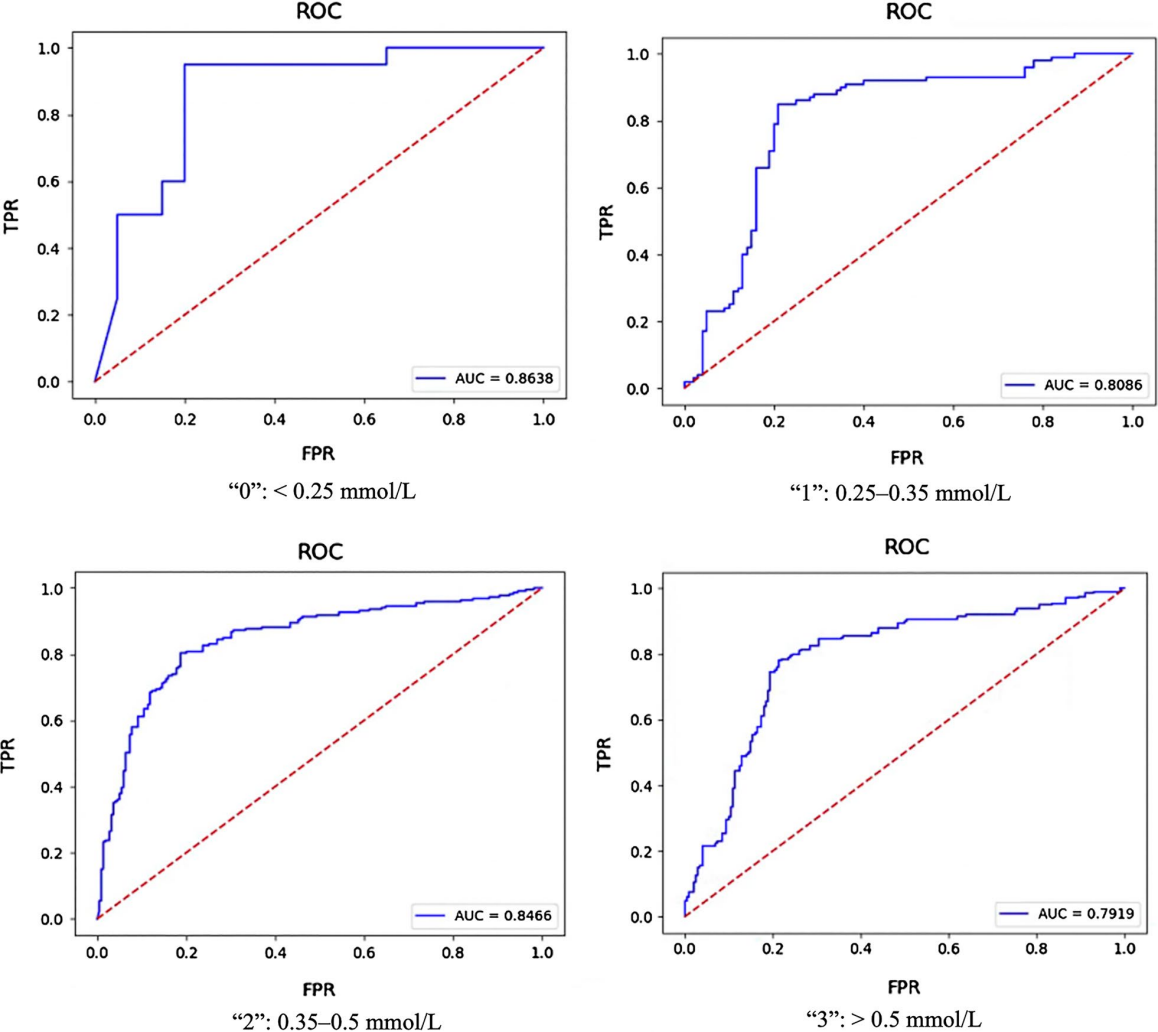
ROC curves and AUC for each classifier

When externally validating the application capability of recommend shallow neural network classifier model, the validation results in dataset 2, a recent and new cohort of patient admitted in 2019, are list in Table 3. The F1 score (80.00%, 80.46%, 80.37%, and 78.90% for labels 0, 1, 2, and 3 of the PUMCH ICU database, respectively), and the AUC (0.8638, 0.8086, 0.8466, and 0.7919) for labels 0, 1, 2, and 3 of the PUMCH ICU database, respectively). Figure 3 shows the ROC and AUC of validation results in dataset 2. The external validation results proved that shallow neural network has stable performance for predicting post-filter ionized calcium levels.

**Table 3:** Performance of the recommend shallow neural network classifier models in validation dataset

Labels	AUC	Precision (%)	Recall (%)	F1-score (%)	Accuracy (%)
“0”: < 0.25 mmol/L	0.8638	80.00	80.00	80.00	80.00
“1”: 0.25–0.35 mmol/L	0.8086	80.75	80.50	80.46	80.50
“2”: 0.35–0.5 mmol/L	0.8466	80.37	80.37	80.37	80.36
“3”: > 0.5 mmol/L	0.7919	78.45	78.00	78.90	78.32

Table 4 shows the feature significance measuring by LASSO regression, which represents the importance of the features. By analyzing the importance of the features of the data, we found that PH of replacement fluid has the greatest impact on the model, followed by Value of Citrate, and then 5% NaHCO_3_, AGE, Temperature, and replacement. Fluid and Gender, but in clinical trials, Value of Citrate should be the most influential, followed by PH of replacement fluid and 5% NaHCO_3_, then AGE, Temperature, replacement fluid and Gender, we use LASSO regression to analyze the feature importance of the data. By observing the results, we find that the feature importance of Value of Citrate and PH of replacement fluid is not much different, which is basically consistent with clinical analysis.

**Table 4:** Feature significance by LASSO regression

Gender	Age	Value of citrate	5%NaHCO_3_	PH of replacement fluid	Replacement fluid	Temperature
0.000000000	0.0000000	0.00000000	0.00000000	0.000000000	0.00000000	0.00000000
0.000000000	0.00000000	0.00000000	0.0000000	0.02172580	0.000000000	0.00000000
0.000000000	0.00000000	− 0.03739033	0.0000000	0.05911613	0.000000000	0.00000000
0.000000000	− 0.02880499	− 0.06103975	0.0000000	0.07894143	0.000000000	0.00000000
0.000000000	− 0.10513137	− 0.13510577	0.1056217	0.16482696	0.000000000	0.00000000
0.000000000	− 0.11214836	− 0.14049981	0.1159727	0.17347107	− 0.004691875	0.00000000
0.000000000	− 0.12624543	− 0.14999870	0.1331873	0.18787215	− 0.012946584	− 0.01223604
0.005256983	− 0.13273391	− 0.15501799	0.1405570	0.19460279	− 0.016788690	− 0.01857270

An extended experiments and evaluation were further conducted on MIMIC-III database to verify the feasibility of this study, and the results also demonstrated shallow neural network achieved the best performance among four models. The detailed results are descried in Additional file 1: Appendix II.

### Assessment results on citric acid overdose

A 10-round random selection of 400 records was conducted for each group. We counted the average number of records with citric acid overdoses to evaluate the citric acid overdose distribution in the four groups. The results demonstrated that the lower post-filter ionized calcium level, the greater the number of records showing citric acid overdose. The results are shown in Table 5.

**Table 5:** Citric acid overdose distribution on four patient groups

Post-filter ionized calcium levels	Average number of records of citric acid overdosing	Randomly selected number of records
< 0.25 mmol/L	36	400/10 rounds
0.25–0.35 mmol/L	34	400/10 rounds
0.35–0.5 mmol/L	31	400/10 rounds
> 0.5 mmol/L	21	400/10 rounds

### Adjustment suggestions for regional citrate anticoagulation therapy

According to the prediction results for the post-filter ionized calcium levels, different adjustment suggestions for the citrate pumping rate can be provided to clinicians, as displayed in Table 6.

**Table 6:** Adjustment suggestions on citrate pumping rate

Post-filter ionized calcium levels (mmol/L)	Citrate pumping rate
< 0.25	Reduce 10 mL/h
0.25–0.35	Stay still
0.35–0.5	Increase 10 mL/h
> 0.5	Increase 20 mL/h

By calculating the ratio of the total calcium measured in vitro and free calcium measured in the blood gas, it is possible to identify whether a patient has citric acid poisoning. When the ratio of the total calcium to ionized calcium is greater than 2.5, a citrate intoxication alert should be provided to clinicians. We thus recommend adjusting the pumping of 10% calcium gluconate to maintain the ionized calcium of the artery at 1.0–1.2 mmol/L. The specific adjustments are shown in Table 7.

**Table 7:** Adjustment suggestions on 10% calcium gluconate input rate

Arterial or venous ionized calcium (mmol/L)	10% calcium gluconate input rate
> 1.45	Reduce 1.5 mL/h
1.21–1.45	Reduce 0.8 mL/h
1.00–1.20	Stay still
0.90–1.00	Increase 0.8 mL/h
< 0.9	After static pushing 10 mL, increase 1.5 mL/h

## Discussions

With the continuous deeper integration of computer technology and medical treatments, the application of machine learning methods in clinical medicine has become an important research topic. Regional citrate anticoagulation has different advantages than heparin anticoagulation. First, the risk of systemic bleeding is significantly reduced; second, the frequency of detection is significantly reduced. Finally, and most importantly, the service life of the filter is significantly extended. At present, it is gradually becoming the mainstream anticoagulation model for bedside kidney replacement in critically ill patients. However, as the indicators that must be monitored are more complicated than those for heparin anticoagulation (and simultaneously can be mixed with human factors), the processes for starting anticoagulation and adjusting the citrate dosage (to avoid overdosing that would harm the liver) and calcium gluconate pumping dosage may sometimes vary, i.e., they may be personalized. Therefore, it fits an artificial intelligence algorithm more naturally. According to our knowledge, this article is the first original work to discuss a regional citrate anticoagulation adjustment strategy under the guidance of artificial intelligence; thus, it may be more beneficial to the future of intelligent ICUs.

This article explores the feasibility of machine learning method supported regional citrate anticoagulation that are based on the current mainstream regional citrate anticoagulation guidelines, expert consensus, and clinical operation paths (to a certain extent). It is in line with various renal replacements in current intensive medical treatment processes. Furthermore, two databases, PUMCH ICU and MIMIC III, were used for developing and evaluating the model methods. The results from the overall comparison of the four models show that the classification effect of the shallow neural network is the best.

An increasing number of examples show that neural networks have broad application prospects in the medical field. The neural network structure is like a biological nervous system and is used to simulate interactions between living things and the natural environment. A neural network has a self-learning function, associative storage function, and ability to seek optimized solutions at high speed. Under the modern conditions of a regional citrate anticoagulation treatment, human intervention is getting increasingly less important.

This study has limitations. At present, the PUMCH ICU database is used for master analysis. Although the data collected from MIMIC III database were used for evaluation, due to the differences of clinical environments and patient cohorts, the MIMIC III dataset does not record consistent ranges of the post-filter ionized calcium with the PUMCH ICU database, so we classified it according to the empirical values (< 0.8 mmol/L, 0.8–0.9 mmol/L, and 0.9–1.0 mmol/L), and the results also recommended shallow neural network as the practical implementation model. We plan to complete a perspective multi-center study, and expand the verification set to meet the real-time needs for more accurate results and fine granularity.

In the future, to prevent citric acid overdose during patient treatment, we will continuously work on citrate monitoring, prediction, and adjustment related studies. Our study results can be utilized and further validated in clinical practice to better support the development of high-quality, evidence-informed, clinical practice guidelines.

## Conclusions

In this study, we prototyped machine learning models with adjustable parameters to determine how to adjust the speed of the pump. The results of this study demonstrated the feasibility of machine learning method for monitoring and adjusting local regional citrate anticoagulation, and further provide data-driven decision-making recommendations to clinicians.

## Supplementary Information

**Additional file 1.** **Appendix I.** The key parameters of the four models. **Appendix II.** MIMIC III extended experiments and evaluation.

## Data Availability

PUMCH ICU data containing protected health information were not available to share. MIMIC III database is publicly available.

## Abbreviations

CRRT: Continuous renal replacement therapy
ICU: Intensive care units
SVM: Support vector machine
KDIGO: Kidney disease improving global outcomes
RCA: Regional citrate anticoagulation
