# Supplementary material for: Early warning of citric acid overdose and timely adjustment of regional citrate anticoagulation based on machine learning methods
Source: BMC Med Inform Decis Mak. 2021 Jul 30;21(Suppl 2):126. doi: 10.1186/s12911-021-01489-8 (PMC8323216; doi:10.1186/s12911-021-01489-8)
Supplement: Supplementary file 1 — Additional file 1. Appendix I. The key parameters of the four models. Appendix II. MIMIC III extended experiments and evaluation. [file 12911_2021_1489_MOESM1_ESM.docx]

**Appendix I.** The key parameters of the four models

| **Models** | **Parameters** |
| --- | --- |
| Adaboost | n_estimators=100 |
| SVM | kernel='rbf', class_weight='balanced' |
| XGBoost | learning_rate=0.1, n_estimators=500, max_depth= 6, min_child_weight=1, seed= 0, subsample= 0.8, colsample_bytree= 0.8, gamma= 0, reg_alpha= 0, reg_lambda= 1 |
| Shallow neural network | learning_rate = 0.00015, training_epochs = 3000, batch_size_0 = 50 |

**Appendix II.** MIMIC III extended experiments and evaluation

We conducted an extended experiments and evaluation on the Medical Information Mart for Intensive Care (MIMIC)-III database. According to the same inclusion criteria, 1291 records from 314 patients were collected from the MIMIC-III database; however, the distributions of the post-filter ionized calcium levels are imbalanced as compared with the PUMCH ICU data. For example, there are few observed post-filter ionized calcium levels belonging to the range ‘<0.25 mmol/L’; therefore, according to the MIMIC-III data distribution, we divided the levels into three categories (<0.8 mmol/L, 0.8–0.9 mmol/L, and 0.9-1.0 mmol/L) for model prediction and validation purposes, as shown in Table (a1). For the MIMIC-III dataset, 100 positive samples were taken for each label, and a total of 300 positive samples were obtained.

Table (a1). Classification labels for post-filter ionized calcium prediction (MIMIC-III dataset)

| Labels | Post-filter ionized calcium levels | Number of records |
| --- | --- | --- |
| 0 | < 0.8 mmol/L | 96 |
| 1 | 0.81–0.9 mmol/L | 336 |
| 2 | 0.91–1 mmol/L | 859 |

As listed in Table (a2), extreme gradient boosting achieved the second best F1 score (78.85%, 72.03%, and 78.98% for labels 0, 1, and 2 of the MIMIC-III database, respectively), second only to that of shallow neutral network (88.85%, 77.77%, and78.60% for labels 0, 1, and 2 of the MIMIC-III database, respectively),  The SVM model also performed very well for all three data sets (68.53%, 67.56%, and 75.70% for labels 0, 1, and 2 of the MIMIC-III database, respectively). The adaptive boosting model performed slightly worse (67.69%, 62.03%, and 64.80% for labels 0, 1, and 2 of the MIMIC-III database, respectively) than the above three models. The results show that shallow neutral network model has the best classification effect among the four models.

Table (a2). Extended experiments and evaluation results (MIMIC-III dataset)

| Labels | Models | Precision | Recall | F1-score | Accuracy |
| --- | --- | --- | --- | --- | --- |
| “0”:  <0.8 mmol/L | AdaBoost | 74.24% | 65.62% | 67.69% | 78.69% |
|  | XGBoost | 80.36% | 81.25% | 78.85% | 81.25% |
|  | SVM | 70.51% | 67.36% | 68.53% | 79.13% |
|  | Shallow neural network | **89.83%** | **88.89%** | **88.85%** | **88.89%** |
| “1”  0.8–0.9 mmol/L | AdaBoost | 67.15% | 62.40% | 62.03% | 72.69% |
|  | XGBoost | 72.71% | 68.31% | 72.03% | 76.33% |
|  | SVM | 70.36% | 65.88% | 67.56% | 75.77% |
|  | Shallow neural network | **78.33%** | **77.86%** | **77.77%** | **78.44%** |
| “2”  0.9-1.0 mmol/L | AdaBoost | 67.84% | 63.54% | 64.80% | 76.56% |
|  | XGBoost | 78.83% | 80.21% | **78.98%** | 80.20% |
|  | SVM | 76.54% | 75.00% | 75.70% | 79.29% |
|  | Shallow neural network | **83.03%** | **84.99%** | 78.60% | **84.21%** |
